# Crystal structure of 4-allyl­sulfanyl-1*H*-pyrazolo­[3,4-*d*]pyrimidine

**DOI:** 10.1107/S1600536814018042

**Published:** 2014-08-23

**Authors:** Mohammed El Fal, Youssef Ramli, El Mokhtar Essassi, Mohamed Saadi, Lahcen El Ammari

**Affiliations:** a Laboratoire de Chimie Organique Hétérocyclique URAC 21, Pôle de Compétence Pharmacochimie, Av. Ibn Battouta, BP 1014, Faculté des Sciences, Université Mohammed V-Agdal, Rabat, Morocco; b Laboratoire National de Contrôle des Médicaments, D M P, Ministère de la Santé, Madinat Al Irnane, BP 6206, Rabat, Morocco; c Laboratoire de Chimie du Solide Appliquée, Faculté des Sciences, Université Mohammed V-Agdal, Avenue Ibn Battouta, BP 1014, Rabat, Morocco

**Keywords:** crystal structure, pyrazolo­pyrimidine, thio­pyrazolo­pyrimidine, disorder

## Abstract

In the title compound, C_8_H_8_N_4_S, the pyrazolo­[3,4-*d*]pyrimidine ring system is essentially planar, with a maximum deviation from the mean plane of 0.025 (3) Å. The allyl group is disordered over two sites in a 0.512 (6):0.488 (6) ratio. In the crystal, mol­ecules are linked by pairs of N—H⋯N hydrogen bonds, forming inversion dimers with an *R*_2_^2^(8) graph-set motif.

## Related literature   

Anti­viral, anti­mycobacterial and anti­cancer properties of pyrazolo­[3,4-*d*]pyrimidine-4(5*H*)-thione derivatives are described, respectively, by Yuan *et al.* (2013 ▶), Ballell *et al.* (2007 ▶) and Rashad *et al.* (2011 ▶), and Alsubari *et al.* (2011 ▶). A similar structure, namely 4-benzyl­sulfanyl-1*H*-pyrazolo­[3,4-*d*]pyrimidine, is reported by El Fal *et al.* (2013 ▶).
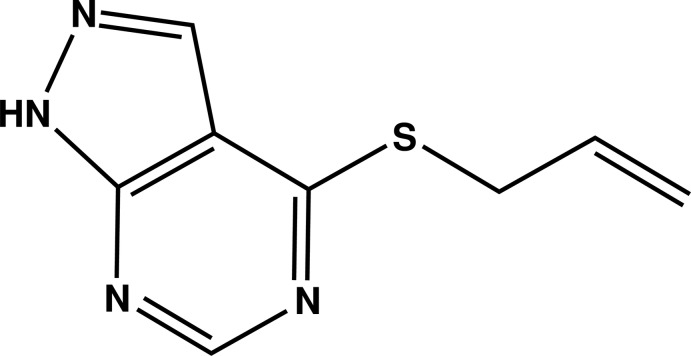


## Experimental   

### Crystal data  

C_8_H_8_N_4_S
*M* *_r_* = 192.24
Orthorhombic, 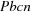

*a* = 18.537 (6) Å
*b* = 5.1997 (17) Å
*c* = 19.059 (7) Å
*V* = 1837.0 (11) Å^3^
*Z* = 8
Mo *K*α radiation
μ = 0.31 mm^−1^
*T* = 296 K
0.39 × 0.34 × 0.29 mm

### Data collection  

Bruker X8 APEX diffractometer
Absorption correction: multi-scan (*SADABS*; Bruker, 2009 ▶) *T*_min_ = 0.641, *T*_max_ = 0.746
20928 measured reflections
2189 independent reflections
1093 reflections with *I* > 2σ(*I*)
*R*_int_ = 0.068

### Refinement  

*R*[*F* ^2^ > 2σ(*F* ^2^)] = 0.056
*wR*(*F* ^2^) = 0.181
*S* = 1.02
2189 reflections
128 parameters
6 restraints
H-atom parameters constrained
Δρ_max_ = 0.28 e Å^−3^
Δρ_min_ = −0.31 e Å^−3^

### 

Data collection: *APEX2* (Bruker, 2009 ▶); cell refinement: *SAINT-Plus* (Bruker, 2009 ▶); data reduction: *SAINT-Plus*; program(s) used to solve structure: *SHELXS97* (Sheldrick, 2008 ▶); program(s) used to refine structure: *SHELXL97* (Sheldrick, 2008 ▶); molecular graphics: *ORTEP-3 for Windows* (Farrugia, 2012 ▶); software used to prepare material for publication: *PLATON* (Spek, 2009 ▶) and *publCIF* (Westrip, 2010 ▶).

## Supplementary Material

Crystal structure: contains datablock(s) I. DOI: 10.1107/S1600536814018042/is5372sup1.cif

Structure factors: contains datablock(s) I. DOI: 10.1107/S1600536814018042/is5372Isup2.hkl

Supporting information file. DOI: 10.1107/S1600536814018042/is5372Isup3.cml

**Fig. 1.**
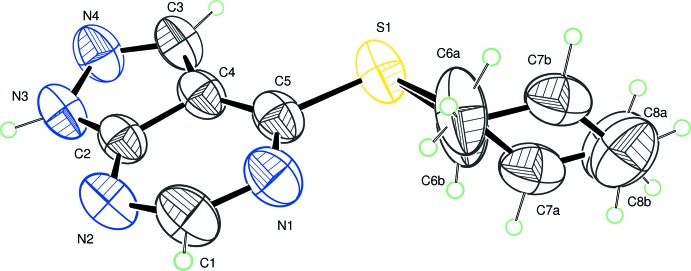
Molecular structure of the title compound with the atom-labelling scheme. Displacement ellipsoids are drawn at the 50% probability level. H atoms are represented as small circles.

**Fig. 2.**
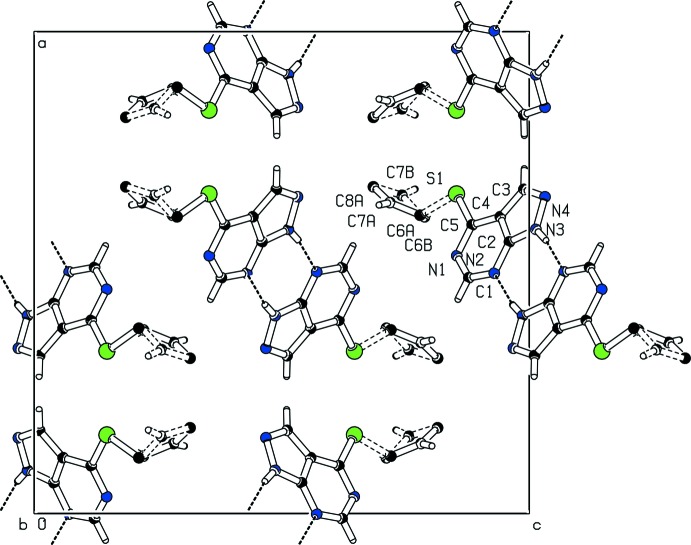
Packing diagram of the title compound viewed along the b axis, showing molecules linked through N3–H3N···N2 hydrogen bond (dashed lines).

. DOI: 10.1107/S1600536814018042/is5372fig1.tif
Mol­ecular structure of the title compound with the atom-labelling scheme. Displacement ellipsoids are drawn at the 50% probability level. H atoms are represented as small circles.

b N . DOI: 10.1107/S1600536814018042/is5372fig2.tif
Packing diagram of the title compound viewed along the *b* axis, showing mol­ecules linked through N3–H3*N*⋯N2 hydrogen bond (dashed lines).

CCDC reference: 1018090

Additional supporting information:  crystallographic information; 3D view; checkCIF report

## Figures and Tables

**Table 1:** Hydrogen-bond geometry (Å, °)

*D*—H⋯*A*	*D*—H	H⋯*A*	*D*⋯*A*	*D*—H⋯*A*
N3—H3*N*⋯N2^i^	0.86	2.09	2.940 (4)	172

Symmetry code: (i) 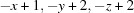.

## supplementary crystallographic information

### S1. Experimental

1*H*,5*H*-pyrazolo[3,4-*d*]pyrimidine-4-thione (0.5 g, 3.29 mmol), allyl bromide (0.5 ml, 5.70 mmol) and potassium carbonate (0.64 g, 4.8 mmol) with a catalytic amount of tetra-n-butylammonium bromide were stirred in DMF (15 ml) for 72 h. The solid obtained was removed by filtration and the solvent evaporated under vacuum. The solid product was purified by recrystallization from ethanol to afford yellow crystals in 55% yield.

### S2. Refinement

The allyl group is disordered over two sites with refined occupancies of 0.512 (6) and 0.488 (6). For the disordered group, tight distance restraints of S—C = 1.753 (2) Å, C—C = 1.453 (2) Å and C═C = 1.287 (2) Å, and constraint of same displacement parameters were applied. The H atoms were located in a difference map and treated as riding with C—H = 0.93 Å (aromatic), C—H = 0.97 Å (methylene) and N—H = 0.86 Å (N–H), and with *U*_iso_(H) = 1.2*U*_eq_(C,N).

### Crystal data

**Table:**

C_8_H_8_N_4_S	*F*(000) = 800
*M**_r_* = 192.24	*D*_x_ = 1.390 Mg m^−^^3^
Orthorhombic, *P**b**c**n*	Mo *K*α radiation, λ = 0.71073 Å
Hall symbol: -p 2n 2ab	Cell parameters from 2189 reflections
*a* = 18.537 (6) Å	θ = 2.4–27.9°
*b* = 5.1997 (17) Å	µ = 0.31 mm^−^^1^
*c* = 19.059 (7) Å	*T* = 296 K
*V* = 1837.0 (11) Å^3^	Block, yellow
*Z* = 8	0.39 × 0.34 × 0.29 mm

### Data collection

**Table:**

Bruker X8 APEX diffractometer	2189 independent reflections
Radiation source: fine-focus sealed tube	1093 reflections with *I* > 2σ(*I*)
Graphite monochromator	*R*_int_ = 0.068
φ and ω scans	θ_max_ = 27.9°, θ_min_ = 2.4°
Absorption correction: multi-scan (*SADABS*; Bruker, 2009)	*h* = −15→24
*T*_min_ = 0.641, *T*_max_ = 0.746	*k* = −6→6
20928 measured reflections	*l* = −24→24

### Refinement

**Table:**

Refinement on *F*^2^	Primary atom site location: structure-invariant direct methods
Least-squares matrix: full	Secondary atom site location: difference Fourier map
*R*[*F*^2^ > 2σ(*F*^2^)] = 0.056	Hydrogen site location: inferred from neighbouring sites
*wR*(*F*^2^) = 0.181	H-atom parameters constrained
*S* = 1.02	*w* = 1/[σ^2^(*F*_o_^2^) + (0.0784*P*)^2^ + 0.5307*P*] where *P* = (*F*_o_^2^ + 2*F*_c_^2^)/3
2189 reflections	(Δ/σ)_max_ < 0.001
128 parameters	Δρ_max_ = 0.28 e Å^−^^3^
6 restraints	Δρ_min_ = −0.31 e Å^−^^3^

### Special details

**Table:**

Geometry. All e.s.d.'s (except the e.s.d. in the dihedral angle between two l.s. planes) are estimated using the full covariance matrix. The cell e.s.d.'s are taken into account individually in the estimation of e.s.d.'s in distances, angles and torsion angles; correlations between e.s.d.'s in cell parameters are only used when they are defined by crystal symmetry. An approximate (isotropic) treatment of cell e.s.d.'s is used for estimating e.s.d.'s involving l.s. planes.
Refinement. Refinement of *F*^2^ against all reflections. The weighted *R*-factor *wR* and goodness of fit *S* are based on *F*^2^, conventional *R*-factors *R* are based on *F*, with *F* set to zero for negative *F*^2^. The threshold expression of *F*^2^ > σ(*F*^2^) is used only for calculating *R*-factors(gt) *etc*. and is not relevant to the choice of reflections for refinement. *R*-factors based on *F*^2^ are statistically about twice as large as those based on *F*, and *R*- factors based on all data will be even larger.

### Fractional atomic coordinates and isotropic or equivalent isotropic displacement parameters (Å^2^)

**Table:**

	*x*	*y*	*z*	*U*_iso_*/*U*_eq_	Occ. (<1)
S1	0.66313 (5)	0.15662 (17)	0.85306 (5)	0.0890 (4)	
N1	0.53488 (15)	0.3882 (5)	0.85359 (15)	0.0819 (8)	
N2	0.49883 (12)	0.7352 (5)	0.92947 (15)	0.0759 (8)	
N3	0.58954 (12)	0.8446 (5)	1.01342 (15)	0.0760 (8)	
H3N	0.5667	0.9680	1.0337	0.091*	
N4	0.65714 (13)	0.7630 (6)	1.03189 (17)	0.0867 (9)	
C1	0.48931 (17)	0.5665 (7)	0.8781 (2)	0.0887 (10)	
H1	0.4446	0.5730	0.8561	0.106*	
C2	0.56370 (15)	0.7078 (5)	0.96002 (17)	0.0639 (8)	
C3	0.67252 (15)	0.5738 (7)	0.98923 (19)	0.0787 (9)	
H3	0.7154	0.4812	0.9902	0.094*	
C4	0.61592 (14)	0.5282 (5)	0.94180 (17)	0.0654 (8)	
C5	0.59890 (15)	0.3712 (5)	0.88464 (18)	0.0710 (9)	
C6A	0.6154 (5)	−0.011 (2)	0.7877 (4)	0.1188 (19)	0.512 (6)
H6A1	0.6140	−0.1915	0.7999	0.143*	0.512 (6)
H6A2	0.5660	0.0515	0.7869	0.143*	0.512 (6)
C7A	0.6458 (4)	0.0154 (16)	0.7178 (5)	0.0919 (15)	0.512 (6)
H7A	0.6385	0.1722	0.6954	0.110*	0.512 (6)
C8A	0.682 (3)	−0.155 (6)	0.6835 (9)	0.121 (3)	0.512 (6)
H8A1	0.6906	−0.3151	0.7035	0.145*	0.512 (6)
H8A2	0.6989	−0.1179	0.6388	0.145*	0.512 (6)
C6B	0.6188 (5)	0.019 (2)	0.7806 (4)	0.1188 (19)	0.488 (6)
H6B1	0.5749	−0.0628	0.7973	0.143*	0.488 (6)
H6B2	0.6046	0.1563	0.7491	0.143*	0.488 (6)
C7B	0.6598 (4)	−0.1692 (16)	0.7409 (5)	0.0919 (15)	0.488 (6)
H7B	0.6757	−0.3111	0.7663	0.110*	0.488 (6)
C8B	0.677 (3)	−0.168 (7)	0.6756 (10)	0.121 (3)	0.488 (6)
H8B1	0.6634	−0.0313	0.6470	0.145*	0.488 (6)
H8B2	0.7042	−0.3024	0.6569	0.145*	0.488 (6)

### Atomic displacement parameters (Å^2^)

**Table:**

	*U*^11^	*U*^22^	*U*^33^	*U*^12^	*U*^13^	*U*^23^
S1	0.0830 (6)	0.0671 (6)	0.1169 (9)	0.0018 (4)	0.0180 (5)	−0.0160 (5)
N1	0.0756 (16)	0.0624 (16)	0.108 (2)	−0.0024 (13)	−0.0034 (15)	−0.0048 (15)
N2	0.0599 (14)	0.0576 (15)	0.110 (2)	0.0017 (11)	−0.0054 (14)	−0.0014 (16)
N3	0.0589 (13)	0.0613 (15)	0.108 (2)	0.0089 (11)	0.0049 (14)	−0.0078 (15)
N4	0.0621 (15)	0.083 (2)	0.115 (2)	0.0106 (13)	−0.0057 (14)	−0.0153 (18)
C1	0.0683 (18)	0.073 (2)	0.124 (3)	−0.0002 (17)	−0.0086 (19)	0.000 (2)
C2	0.0604 (15)	0.0472 (15)	0.084 (2)	−0.0016 (12)	0.0065 (15)	0.0029 (15)
C3	0.0630 (17)	0.069 (2)	0.104 (3)	0.0116 (14)	0.0028 (17)	−0.009 (2)
C4	0.0584 (15)	0.0511 (16)	0.087 (2)	−0.0005 (12)	0.0069 (15)	0.0023 (16)
C5	0.0704 (18)	0.0502 (17)	0.092 (2)	−0.0041 (14)	0.0122 (17)	0.0058 (17)
C6A	0.103 (3)	0.098 (4)	0.155 (4)	−0.023 (3)	0.038 (3)	−0.054 (3)
C7A	0.094 (3)	0.076 (4)	0.106 (4)	−0.009 (3)	−0.019 (3)	0.018 (3)
C8A	0.102 (6)	0.167 (5)	0.093 (5)	−0.012 (4)	−0.007 (6)	−0.010 (5)
C6B	0.103 (3)	0.098 (4)	0.155 (4)	−0.023 (3)	0.038 (3)	−0.054 (3)
C7B	0.094 (3)	0.076 (4)	0.106 (4)	−0.009 (3)	−0.019 (3)	0.018 (3)
C8B	0.102 (6)	0.167 (5)	0.093 (5)	−0.012 (4)	−0.007 (6)	−0.010 (5)

### Geometric parameters (Å, º)

**Table:**

S1—C5	1.739 (3)	C4—C5	1.397 (4)
S1—C6A	1.759 (2)	C6A—C7A	1.452 (2)
S1—C6B	1.759 (2)	C6A—H6A1	0.9700
N1—C5	1.329 (4)	C6A—H6A2	0.9700
N1—C1	1.339 (4)	C7A—C8A	1.287 (2)
N2—C1	1.326 (4)	C7A—H7A	0.9300
N2—C2	1.344 (4)	C8A—H8A1	0.9300
N3—C2	1.331 (4)	C8A—H8A2	0.9300
N3—N4	1.369 (3)	C6B—C7B	1.453 (2)
N3—H3N	0.8600	C6B—H6B1	0.9700
N4—C3	1.308 (4)	C6B—H6B2	0.9700
C1—H1	0.9300	C7B—C8B	1.287 (2)
C2—C4	1.389 (4)	C7B—H7B	0.9300
C3—C4	1.405 (4)	C8B—H8B1	0.9300
C3—H3	0.9300	C8B—H8B2	0.9300
			
C5—S1—C6A	102.6 (4)	C7A—C6A—H6A1	108.7
C5—S1—C6B	102.3 (4)	S1—C6A—H6A1	108.7
C5—N1—C1	117.0 (3)	C7A—C6A—H6A2	108.7
C1—N2—C2	111.6 (3)	S1—C6A—H6A2	108.7
C2—N3—N4	111.1 (3)	H6A1—C6A—H6A2	107.6
C2—N3—H3N	124.4	C8A—C7A—C6A	127.1 (11)
N4—N3—H3N	124.4	C8A—C7A—H7A	116.5
C3—N4—N3	105.8 (3)	C6A—C7A—H7A	116.5
N2—C1—N1	129.2 (3)	C7A—C8A—H8A1	120.0
N2—C1—H1	115.4	C7A—C8A—H8A2	120.0
N1—C1—H1	115.4	H8A1—C8A—H8A2	120.0
N3—C2—N2	126.7 (3)	C7B—C6B—S1	116.0 (7)
N3—C2—C4	107.4 (3)	C7B—C6B—H6B1	108.3
N2—C2—C4	125.9 (3)	S1—C6B—H6B1	108.3
N4—C3—C4	111.4 (3)	C7B—C6B—H6B2	108.3
N4—C3—H3	124.3	S1—C6B—H6B2	108.3
C4—C3—H3	124.3	H6B1—C6B—H6B2	107.4
C2—C4—C5	115.5 (3)	C8B—C7B—C6B	129.2 (10)
C2—C4—C3	104.2 (3)	C8B—C7B—H7B	115.4
C5—C4—C3	140.2 (3)	C6B—C7B—H7B	115.4
N1—C5—C4	120.7 (3)	C7B—C8B—H8B1	120.0
N1—C5—S1	120.0 (3)	C7B—C8B—H8B2	120.0
C4—C5—S1	119.3 (2)	H8B1—C8B—H8B2	120.0
C7A—C6A—S1	114.1 (6)		

### Hydrogen-bond geometry (Å, º)

**Table:**

*D*—H···*A*	*D*—H	H···*A*	*D*···*A*	*D*—H···*A*
N3—H3*N*···N2^i^	0.86	2.09	2.940 (4)	172

Symmetry code: (i) −*x*+1, −*y*+2, −*z*+2.
