# Supplementary figures and images for: Crystal structure of 4-allyl­sulfanyl-1H-pyrazolo­[3,4-d]pyrimidine
Source: Acta Crystallogr Sect E Struct Rep Online. 2014 Aug 23;70(Pt 9):o1038. doi: 10.1107/S1600536814018042 (PMC4186177; doi:10.1107/S1600536814018042)

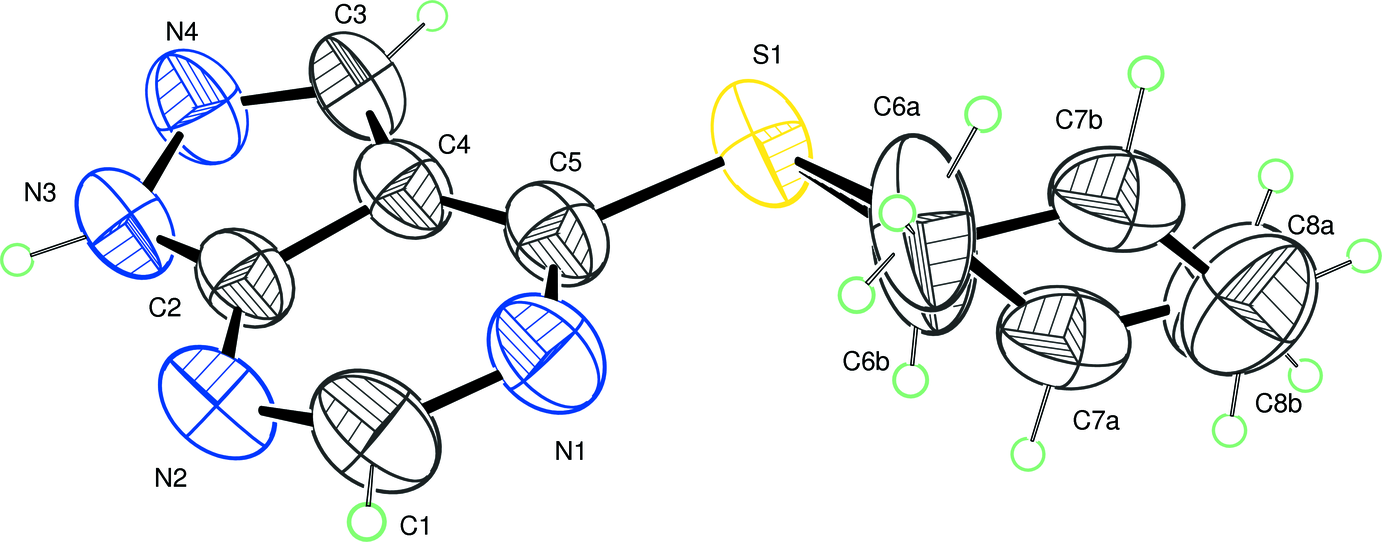

Supplement: Supplementary file 4 [file e-70-o1038-fig1.tif]

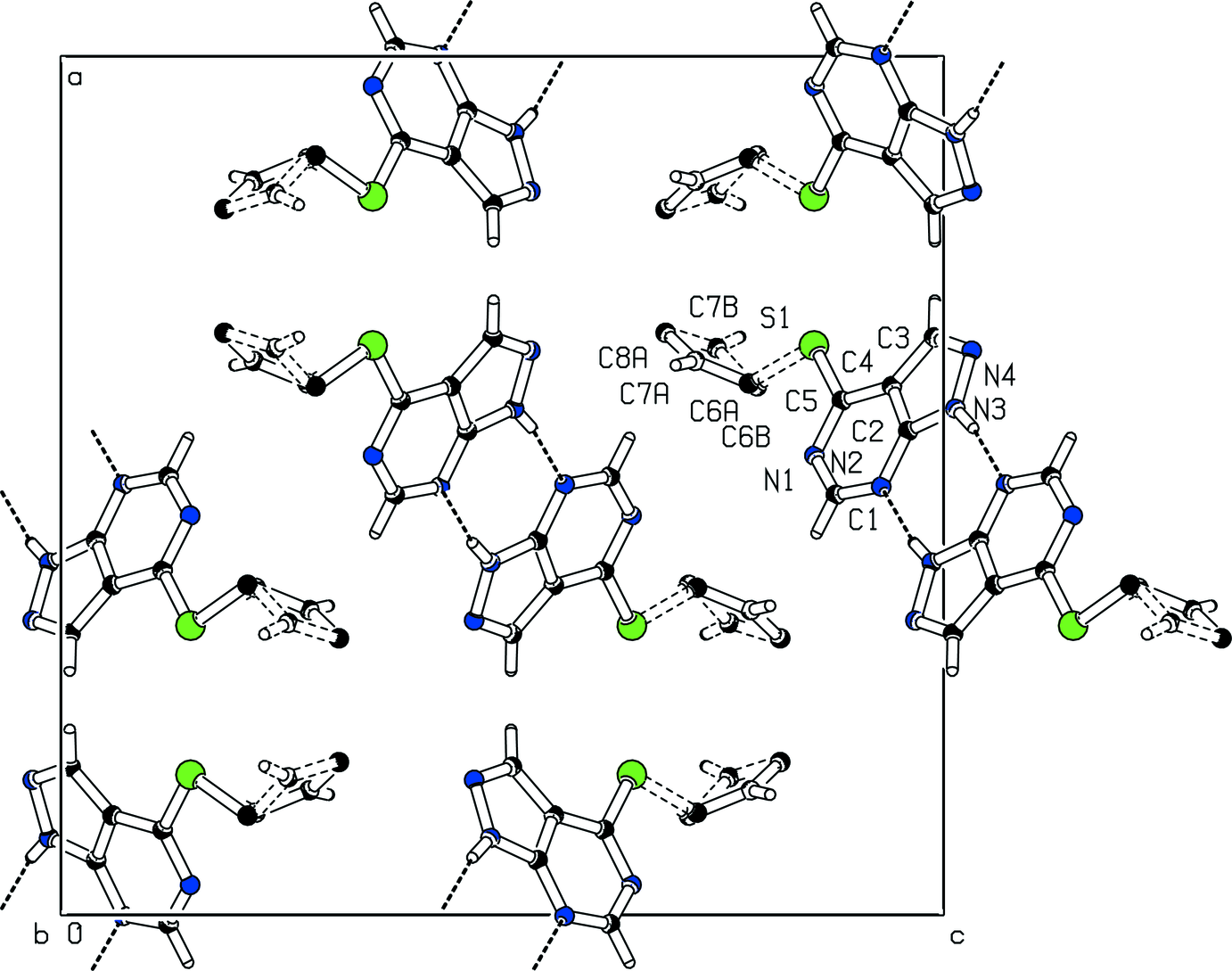

Supplement: Supplementary file 5 [file e-70-o1038-fig2.tif]
